# [Expression of Concern] 5-Azacytidine inhibits the proliferation of bladder cancer cells via reversal of the aberrant hypermethylation of the hepaCAM gene

**DOI:** 10.3892/or.2026.9108

**Published:** 2026-03-30

**Authors:** Xiaorong Wang, E. Chen, Xue Yang, Yin Wang, Zhen Quan, Xiaohou Wu, Chunli Luo

Oncol Rep 35: 1375–1384, 2016; DOI: 10.3892/or.2015.4492

Following the publication of the above paper, it was drawn to the Editor's attention by a concerned reader that, regarding the immunohistochemical images shown in Fig. 1A on p. 1377, the ‘DNMT3A’ and ‘DNMT3B’ images for the ‘BN’ row of data contained an overlapping section, such that date which were intended to show the results from differently performed experiments had apparently been derived from the same original source. In addition, upon performing an independent analysis of the data in this paper in the Editorial Office, it came to light that the same data had been included for the Petri dish images in Fig. 3B on p. 1382 for the DMSO experiments with the T24 and EJ cell lines, albeit the EJ image had been rotated through 90°.

The authors were contacted by the Editorial Office to offer an explanation for this apparent duplication of data within these figures; however, up to this time, no response from them has been forthcoming. Owing to the fact that the Editorial Office has been made aware of potential issues surrounding the scientific integrity of this paper, we are issuing an Expression of Concern to notify readers of this potential problem while the Editorial Office continues to investigate this matter further.

